# A Diagnostic Model With IgM Autoantibodies and Carcinoembryonic Antigen for Early Detection of Lung Adenocarcinoma

**DOI:** 10.3389/fimmu.2021.728853

**Published:** 2022-01-24

**Authors:** Xue Zhang, Jiaqi Li, Yulin Wang, Man Liu, Fenghui Liu, Xiuzhi Zhang, Lu Pei, Tingting Wang, Di Jiang, Xiao Wang, Jianying Zhang, Liping Dai

**Affiliations:** ^1^ Henan Institute of Medical and Pharmaceutical Sciences & School of Basic Medical Sciences, Academy of Medical Science, Zhengzhou University, Zhengzhou, China; ^2^ Henan Key Laboratory of Tumor Epidemiology & Henan Key Medical Laboratory of Tumor Molecular Biomarkers, Zhengzhou University, Zhengzhou, China; ^3^ Department of Respiratory and Sleep Medicine in the First Affiliated Hospital, Zhengzhou University, Zhengzhou, China; ^4^ Department of Pathology, Henan Medical College, Zhengzhou, China; ^5^ Department of Clinical Laboratory, Zhengzhou Hospital of Traditional Chinese Medicine, Zhengzhou, China; ^6^ Department of Clinical Laboratory, Fuwai Central China Cardiovascular Hospital, Zhengzhou, China

**Keywords:** lung adenocarcinoma (LUAD), IgM autoantibody, carcinoembryonic antigen (CEA), protein array, cancer driver gene, diagnostic model

## Abstract

Immunoglobulin M (IgM) autoantibodies, as the early appearing antibodies in humoral immunity when stimulated by antigens, might be excellent biomarkers for the early detection of lung cancer (LC). We aimed to develop a multi-analyte integrative model combining IgM autoantibodies and a traditional tumor biomarker that could be a valuable and powerful auxiliary diagnostic tool and might improve the accuracy of early detection of lung adenocarcinoma (LUAD). A customized protein array based on cancer driver genes was constructed and applied in the discovery cohort consisting of 68 LUAD patients and 68 normal controls (NCs); 31 differentially expressed IgM autoantibodies were identified. The top 5 candidate IgM autoantibodies [based on the area under the receiver operating characteristic curve (AUC) ranking], namely, TSHR, ERBB2, survivin, PIK3CA, and JAK2, were validated in the validation cohort using enzyme-linked immunosorbent assay (ELISA), which included 147 LUAD samples, 72 lung squamous cell carcinoma (LUSC) samples, 44 small cell lung carcinoma (SCLC) samples, and 147 NCs. These indicators presented diagnostic capacity for LUAD, with AUCs of 0.599, 0.613, 0.579, 0.601, and 0.633, respectively (*p* < 0.05). However, none of them showed a significant difference between the SCLC and NC groups, and only the IgM autoantibody against JAK2 showed a higher expression in LUSC than in NC (*p* = 0.046). Through logistic regression analysis, with the five IgM autoantibodies and carcinoembryonic antigen (CEA), one diagnostic model was constructed for LUAD. The model yielded an AUC of 0.827 (sensitivity = 56.63%, specificity = 93.98%). The diagnostic efficiency was superior to that of either CEA (AUC = 0.692) or IgM autoantibodies alone (AUC = 0.698). Notably, the accuracy of this model in early-stage LUAD reached 83.02%. In conclusion, we discovered and identified five novel IgM indicators and developed a multi-analyte model combining IgM autoantibodies and CEA, which could be a valuable and powerful auxiliary diagnostic tool and might improve the accuracy of early detection of LUAD.

## Introduction

With estimates of 2.2 million new cases and 1.8 million deaths, lung cancer (LC) is the second most common cancer and the leading cause of cancer death worldwide, approximately accounting for one-tenth (11.4%) of cancer occurrence and one-fifth (18.0%) of cancer deaths (1). The 5-year survival rate is 57% for patients with localized tumors, while this decreased to 5% for patients at the metastatic stage (2), which indicates that the high mortality rate of LC is closely related to cancer stage. Traditionally, LC is classified into non-small cell lung carcinoma (NSCLC) and small cell lung carcinoma (SCLC). There are two major types of NSCLC: lung adenocarcinoma (LUAD) and lung squamous cell carcinoma (LUSC) (3). LUAD, as the most common lung malignancy, is frequently found in women and non-smokers (3, 4). A large number of studies have shown that, if LC patients with adenocarcinoma *in situ* (AIS) and minimally invasive adenocarcinoma (MIA) could undergo radical surgery, their 5-year disease-free survival rate may approach 100% (5). Therefore, the early diagnosis and treatment of LUAD are essential to reduce the mortality of LC.

Currently, low-dose spiral CT (LDCT) and pathological tissue biopsy are used to screen and detect LC patients clinically, but the former has high false-positive rates and the latter is traumatic, which causes some excessive diagnosis, unnecessary tests, invasive procedures, and, rarely, radiation-induced cancers (6). In recent years, serological biomarkers have received widespread attention because of their advantages, such as being simple, noninvasive, and easily accepted by patients (7, 8). Traditional serum tumor markers had been used in the auxiliary diagnosis of cancers in clinical practice, such as carcinoembryonic antigen (CEA), cancer antigen 125 (CA-125), and cytokeratin-19 fragment (CYFRA 21-1), but their diagnostic ability was limited by their unsatisfactory:: sensitivity and specificity for LC (9, 10).

Tumors are the products of the malignant transformation of normal cells, which are characterized by continuous proliferation and metastasis in the body. The prominent feature of tumor cells in immunology is the appearance of certain tumor-associated antigens that are not visible or have low expressions in normal cells of the same type (11). Due to the presence of tumor-associated antigens, it is bound to be recognized by the body’s immune system and thus stimulate adaptive immune responses, including cellular immunity and humoral immunity (12, 13). Immunoglobulin G (IgG) and immunoglobulin M (IgM) autoantibodies are produced as an important part of humoral immunity and are secreted into the blood. Recent researches have provided substantial evidence that patients with cancers could develop humoral immune response and then produce autoantibodies in the early stage even before cancer diagnosis (14, 15). Therefore, as the primary and secondary response products, IgM and IgG autoantibodies have great potential as early diagnostic indicators of LC. Related studies on IgG autoantibodies in the early diagnosis of LC have made admirable progress (16–18). However, studies regarding IgM autoantibodies are limited. Thus, more research is needed to provide evidence for IgM as an earlier indicator for discriminating LC patients and normal individuals.

In the present study, we aimed to screen valuable IgM autoantibody indicators for LUAD by protein array and verify them in another sample cohort with enzyme-linked immunosorbent assay (ELISA). Ultimately, the five candidate IgM autoantibodies and CEA were integrated to construct a diagnostic model to improve the diagnostic efficiency for LUAD. The diagnostic model might be able to improve the treatment status of LC patients and increase their survival rate.

## Materials and Methods

### Study Population and Serum Collection

All serum samples included in this study were obtained from the Specimen Biobank in Henan Key Medical Laboratory of Tumor Molecular Biomarkers collected from a provincial hospital in Zhengzhou, Henan Province, China, between 2016 and 2019. Two independent sample cohorts (a discovery cohort and a validation cohort) were used in this research. The discovery cohort consisted of 68 LUAD patients (LUADs) and 68 normal controls (NCs) matched by gender and age. In addition, 147 LUADs, 147 matched NCs, 72 LUSC patients, and 44 SCLC patients were included in the validation cohort. The blood samples of all LC patients were drawn upon their first diagnosis without any other cancers, antitumor treatment, and autoimmune diseases. All NCs were individuals who had a health checkup without history of cancer, pulmonary diseases, and autoimmune diseases. The sera were extracted and stored according to standard protocols (19). The study was approved by the Medical Ethics Committee of Zhengzhou University, and all the patients and NCs signed an informed consent before their participation in the study.

The serum CEA test results were provided by the laboratory of the hospital. It was obtained using the MODULARE70 automatic analyzer and supporting kits produced by Roche in Switzerland. The principle was electrochemiluminescence. The experimental operations were carried out by professional and technical personnel. Moreover, the results were released after inspection by experienced laboratory physicians.

### Human Protein Array Assay

The human protein array assay was commissioned to BC Biotechnology Co., Ltd. (Foshan, China) based on the conception of our laboratory. The protein chip contained 154 recombinant proteins or protein fragments, 11 of which (CIP2A/p90, c-Myc, cyclin B1, IMP1, IMP2, IMP3, RalA, RBM39, YWHAZ, and two fragments of survivin), with potential diagnostic value, were reported in our previous research (20, 21); the other 143 were encoded by cancer driver genes. The chips covering the aforementioned proteins were blocked by a blocking buffer [3% albumin from bovine serum (BSA) in a phosphate-buffered saline (PBS) buffer] for 3 h at room temperature. After removing the liquid, 200 μl diluted serum sample (1:50) was added into the corresponding block and incubated on a side swing shaker at 4°C overnight. Then, the chips were washed three times (10 min each time) with 200 μl PBS containing 0.1% Tween 20 (PBST) buffer for each block. The arrays were transferred into an incubation box with 3 ml Cy5-labeled anti-human IgM antibody incubation fluid inside for 1 h in the dark. The wash process was repeated three times with PBST and twice with ddH_2_O. After placing the chips in an array dryer for centrifugal drying, scanning and data extraction were performed using LuxScan 10K-A (CapitalBio Corporation, Beijing, China).

### Data Analysis for Assays Performed on Protein Array

In order to eliminate the deviation caused by the inconsistency of the background value between different samples, the ratio of the foreground value to the background value of each protein (F median/B median) was calculated, namely, the signal-to-noise ratio (SNR). Statistical analyses of the samples were based on the SNR value. The tests for the test samples were repeated 30 times at different time points, different chips, and different positions to evaluate the stability of the operation.

### Selection of Candidate IgM Autoantibodies

Analysis of the receiver operating characteristic (ROC) of each autoantibody in LUADs and NCs was applied based on the SNR value of each samples. Thirty-one IgM autoantibodies were screened and showed higher distinguishing values in LUADs [area under the ROC curve (AUC) > 0.5, *p* < 0.05]. In view of the optimal choice in terms of the number of indicators when constructing the model, we set the top 5 based on AUC rankings (TSHR, ERBB2, survivin, PIK3CA, and JAK2) among the 31 differential autoantibodies as the candidate IgM indicators (**Supplementary Table S1**).

### Enzyme-Linked Immunosorbent Assay

ELISA was used to detect the serum levels of candidate autoantibodies discovered by the protein array. The five purified recombinant proteins (TSHR, ERBB2, survivin, PIK3CA, and JAK2) were coated at an optimal concentration of 0.125 μg/ml (50 μl/well) at 4°C overnight. After blocking with 2% BSA buffer (50 μl/well) at 4°C overnight, all serum samples diluted 1:100 were piped into the antigen-coated wells (50 μl/well) and incubated at 37°C for 1 h. Then, the plates were washed three times with PBST. Subsequently, the horseradish peroxidase (HRP)-conjugated anti-human IgM antibody (1:2,000 dilution) or the HRP-conjugated anti-human IgG antibody (1:5,000 dilution) was added into each microplate well (50 μl/well) and incubated at 37°C for 1 h, followed by washing five times with PBST. TMB (3,3′,5,5′-tetramethylbenzidine) substrate solution was used as the detecting reagent (50 μl/well), and the enzyme–substrate reaction was terminated by the addition of a sulfuric acid solution (25 μl/well). Finally, the color change was measured spectrophotometrically at wavelengths of 450 and 620 nm.

The difference of the optical density (OD) values at 450 and 620 nm was applied in further statistical analysis. To ensure the reliability of the serum levels of the IgM autoantibodies, a positive control, a negative control, and two blank controls were used in every plate and the samples of each group were dispersed on the plates.

### Statistical Analysis

Data analysis and visualization was performed using DAVID (Database for Annotation, Visualization, and Integrated Discovery), SPSS Statistics 26.0, GraphPad Prism 8.0, and R-4.0.0 software. The *χ* ^2^ test and Student’s *t*-test were applied to compare the differences in the characteristics between two cohorts. Besides, differences in the levels of autoantibodies in LC patients and NCs were analyzed using the non-parametric Mann–Whitney *U* test. Logistic regression was employed to construct a diagnostic model for LUAD combining autoantibodies and CEA. ROC analysis and the AUC with 95% confidence interval (CI) were used to evaluate the diagnostic performance of the biomarkers and the model. The sensitivity and specificity were determined based on the cutoff value, which was defined as the OD value at the maximum Youden’s index, while specificity is more than 90%. In all tests, *p* < 0.05 (two-tailed) was considered statistically significant.

## Results

### Overall Study Design and Characteristics of the Study Subjects

**Figure 1:**
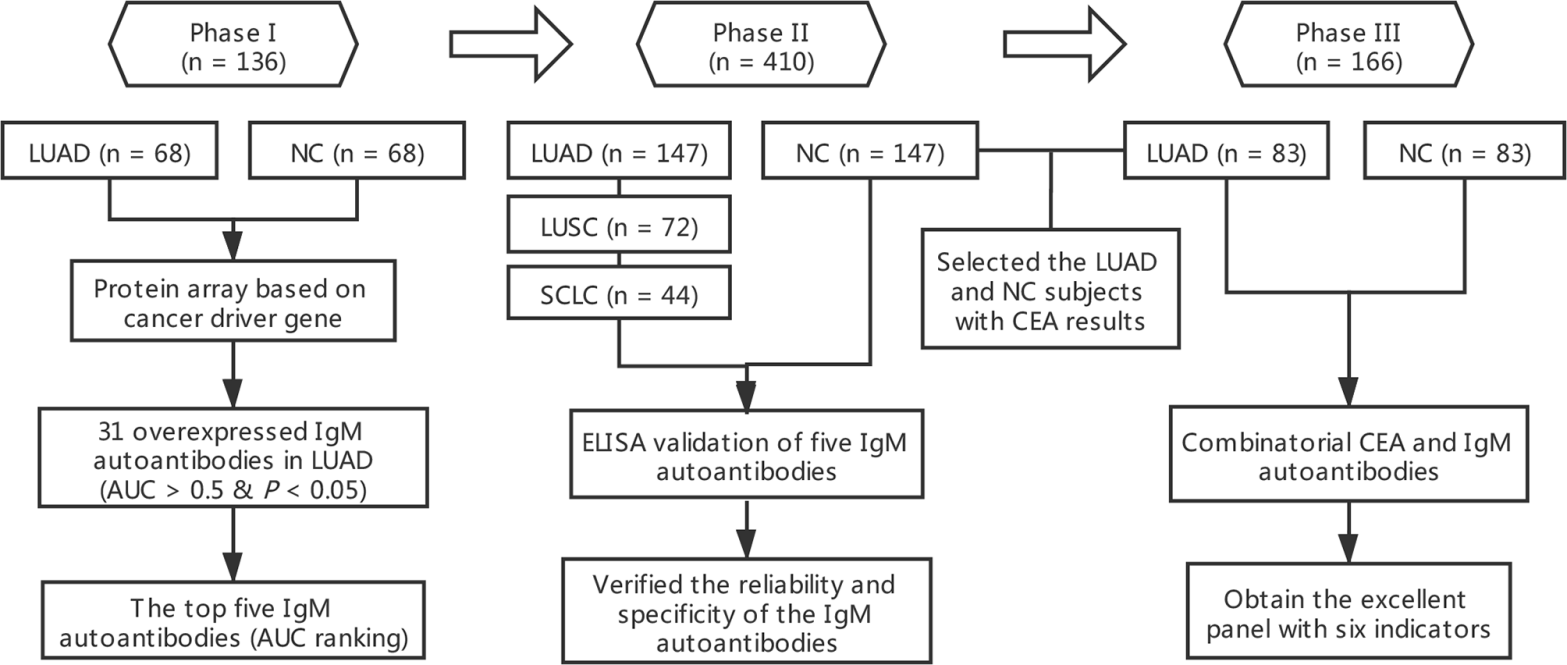
Overall design of this study. AUC, area under the receiver operating characteristic curve; CEA, carcinoembryonic antigen; LUAD, lung adenocarcinoma; LUSC , lung squamous cell carcinoma; NC , normal control; SCLC, small cell lung carcinoma.

In the whole study, we employed a three-phase strategy to identify IgM autoantibodies for the early diagnosis of LUAD and to construct a diagnostic model combining potential IgM autoantibodies and CEA (**Figure 1**). In phase I, the serum samples from the discovery cohort composed of 68 LUADs and 68 matched NCs were individually profiled on the protein array for the screening of IgM autoantibodies. A total of 31 IgM autoantibodies were observed to have higher intensity in the sera of LUADs than in NCs. In phase II, the top 5 (AUC ranking) of the 31 overexpressed IgM autoantibodies were tested in a larger cohort comprising sera from 147 LUADs, 147 matched NCs, 72 LUSC patients, and 44 SCLC patients using indirect ELISA. In phase III, we selected 83 LUADs with CEA results and 83 NCs matched by age and sex from the validation cohort. Based on the CEA and IgM autoantibody results of these populations, a diagnostic model was built, which showed a much improved performance using logistic regression.

The clinical characteristics of all the subjects involved in this study are described in **Table 1**. There were no significant differences in sex, smoking history, TNM stage, status of lymph node metastasis, and distant metastasis in LUADs of the two cohorts.

**Table 1:** Descriptive characteristics of the study population.

Variables	Discovery cohort (*n* = 136)		Validation cohort (*n* = 410)			
	LUAD (*n* = 68)	NC (*n* = 68)	LUAD (*n* = 147)	LUSC (*n* = 72)	SCLC (*n* = 44)	NC (*n* = 147)
**Age (years)**						
Range	35–76	39–78	26–83	26–85	40–78	27–82
Mean ± SD	60.84 ± 8.52	60.58 ± 8.73	57.79 ± 10.79	62.75 ± 10.81	62.82 ± 6.51	57.47 ± 10.71
Sex, *n* (%)						
Male	37 (54.41)	40 (58.82)	75 (51.02)	65 (90.28)	30 (68.18)	75 (51.02)
Female	31 (45.59)	28 (41.18)	72 (48.98)	7 (9.72)	14 (31.82)	72 (48.98)
**Smoking, *n* (%)**						
Yes	20 (29.41)		51 (34.69)	55 (76.39)	28 (63.64)	
No	48 (70.59)		94 (63.95)	17 (23.61)	16 (36.36)	
Unknown			2 (1.36)			
**Stage, *n* (%)**						
I and II	26 (38.24)		49 (33.34)	8 (11.11)		
III and IV	42 (61.76)		90 (61.22)	26 (36.11)	17 (38.64)	
Unknown			8 (5.44)	38 (52.78)	27 (61.36)	
**Lymph node metastasis, *n* (%)**						
Yes	39 (57.35)		79 (53.74)	35 (48.61)	21 (47.73)	
No	20 (29.41)		61 (41.50)	9 (12.50)	2 (4.54)	
Unknown	9 (13.24)		7 (4.76)	28 (38.89)	21 (47.73)	
**Distant metastasis, *n* (%)**						
Yes	25 (36.77)		54 (36.73)	16 (22.22)	16 (36.36)	
No	41 (60.29)		85 (57.82)	22 (30.56)	3 (6.82)	
Unknown	2 (2.94)		8 (5.44)	34 (47.22)	25 (56.82)	
**CEA, *n* (%)**						
>5 ng/ml	20 (29.41)		38 (25.85)	7 (9.72)	5 (11.36)	8 (5.45)
≤5 ng/ml	15 (22.06)		45 (30.61)	33 (45.83)	18 (40.91)	128 (87.07)
Unknown	33 (48.53)		64 (43.54)	32 (44.45)	21 (47.73)	11 (7.48)

CEA, carcinoembryonic antigen; LUAD, lung adenocarcinoma; LUSC, lung squamous cell carcinoma; NC, normal control; SCLC, small cell lung carcinoma; SD, standard deviation.

### Human Protein Array Analysis

**Figure 2:**
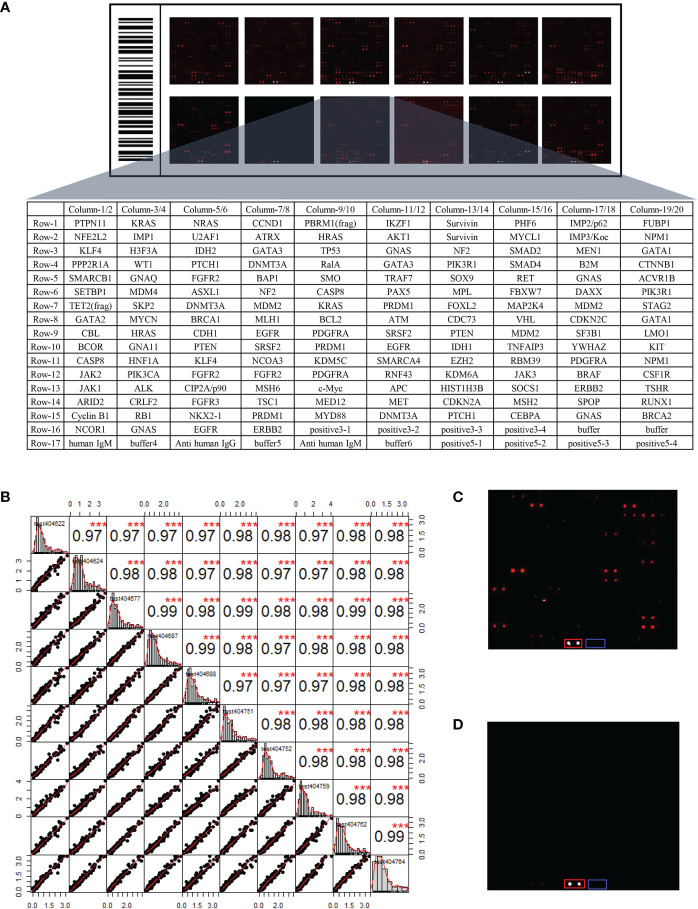
**(A)** Layout of the customized protein array. **(B)** Evaluation of the operational stability of different chips at different time points. The *top right half* represents the correlation results between samples after linear fitting (***p < 0.001), while the *bottom left half* depicts the distribution of the results after linear fitting. **(C)** Protein fluorescence quantification results of a LUAD sample. **(D)** Protein fluorescence quantification results of a NC sample [*red* and *blue frames* highlight the positive control (anti-human IgM) and negative control (buffer)]. LUAD, lung adenocarcinoma; NC, normal control.

In order to identify candidate IgM autoantibodies, we assembled the discovery cohort of 136 serum samples collected from 68 LUADs and 68 matched NCs. The levels of IgM autoantibodies against 154 recombinant proteins were evaluated in the sera using the protein array. As shown in **Figure 2A**, the 154 proteins, the anti-human IgM (positive control), and a buffer (negative control) were arranged in order in the protein chips. The stability test results revealed that the average repeatability between the different batches of chips was 0.98, manifesting that the overall stability was good (**Figure 2B**). Furthermore, as exhibited in the representative results from LUADs and NCs (**Figures 2C** **, D**), the IgM response of LUADs was apparently stronger than that of NCs. The positive and negative controls ensured the credibility of the experimental results.

**Figure 3:**
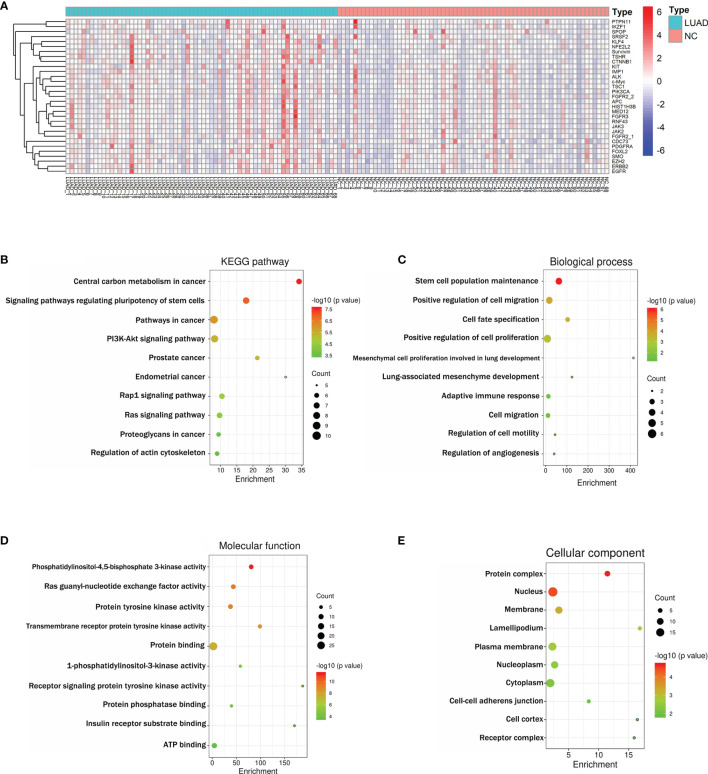
**(A)** Heatmap of the 31 differently expressed IgM autoantibodies between LUAD and NC. **(B)** Schematic representation of the 10 KEGG pathways for the 31 differentially abundant IgM autoantibodies in LUAD (all *p* < 0.001). **(C–E)** Schematic representation of the GO terms for the 31 differentially abundant IgM autoantibodies in LUAD (all *p* < 0.05). GO, Gene Ontology; KEGG , Kyoto Encyclopedia of Genes and Genomes; LUAD , lung adenocarcinoma; NC, normal control. Enrichment (fold enrichment) is defined as the ratio of the two proportions. For example, if 10/22 (i.e., 45.5%) of the input genes involved in “pathways in cancer” and the background information is 393/6,879 genes (i.e., 5.7%) associating with “pathways in cancer”, the fold enrichment is roughly 8 (45.5%/5.7%).

Based on the criteria AUC > 0.5 and *p* < 0.05, 31 of the screened IgM autoantibodies showed higher expressions in LUADs, but much less in NCs (**Figure 3A**). **Supplementary Table S1** illustrates the characteristics and functions of these 31 IgM autoantibodies, which were functionally relevant in tumorigenesis. Subsequently, Kyoto Encyclopedia of Genes and Genomes (KEGG) pathway and Gene Ontology (GO) analyses of the corresponding genes were performed with DAVID. KEGG pathway enrichment analysis showed that the proteins were mostly involved in pathways in cancer, PI3K–Akt signaling pathway and Ras signaling pathway (**Figure 3B**). GO enrichment analysis revealed that the main biological processes involved cell proliferation, cell migration, regulation of angiogenesis, and adaptive immune response (**Figure 3C**), whereas the molecular functions of these proteins were primarily related to protein binding, ATP binding, and receptor signaling protein tyrosine kinase activity (**Figure 3D**). As for the cellular component terms in the GO analysis, the proteins were mainly localized in the nucleus, cytoplasm, and plasma membrane (**Figure 3E**).

### Performance of Five Candidate IgM Autoantibodies in the Discovery and Validation Cohorts

**Figure 4:**
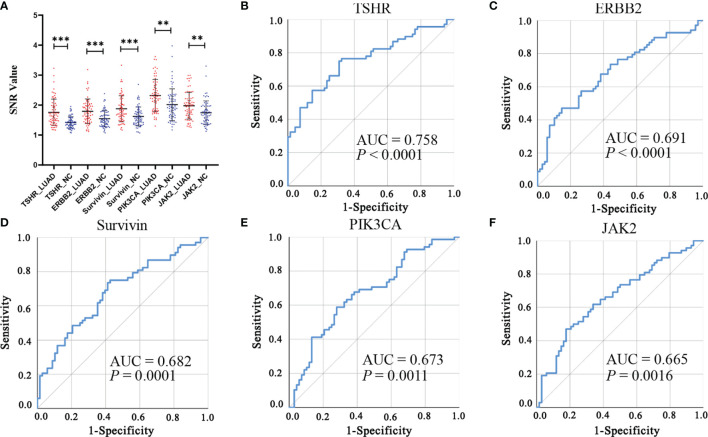
**(A)** Differential expressions of the top 5 (AUC ranking) IgM autoantibodies in the discovery cohort using protein array. ***p < 0.001, **p < 0.01. **(B–F)** ROC analysis of the top 5 IgM autoantibodies in the discovery cohort. AUC, area under the receiver operating characteristic curve; LUAD , lung adenocarcinoma; NC, normal control; ROC, receiver operating characteristic; SNR, signal-to-noise ratio.

**Figure 5:**
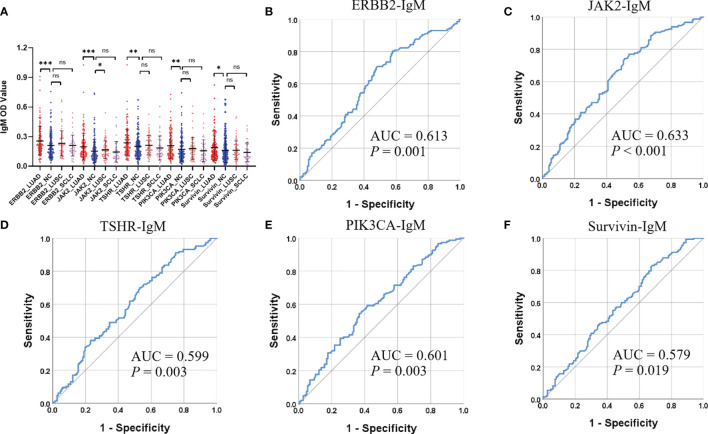
**(A)** Differential expressions of the top 5 (AUC ranking) IgM autoantibodies in the validation cohort using ELISA. ***p < 0.001, **p < 0.01, *p < 0.05, nsp > 0.05. **(B–F)** ROC analysis (LUAD and NC) of the top 5 IgM autoantibodies in the validation cohort. AUC, area under the receiver operating characteristic curve; LUAD, lung adenocarcinoma; LUSC , lung squamous cell carcinoma; NC, normal control; OD , optical density; ROC, receiver operating characteristic; SCLC, small cell lung carcinoma.

Of the 31 aforementioned IgM autoantibodies, we selected the top 5 (AUC ranking) as the candidate biomarkers. In the discovery cohort, the levels of IgM autoantibodies against TSHR, ERBB2, survivin, PIK3CA, and JAK2 (the top 5 in AUC ranking) were significantly higher in LUADs than those in NCs (**Figure 4**). The AUCs of the single IgM autoantibodies ranged from 0.665 to 0.758. Similar results were also found in another independent cohort (validation cohort) that included 147 LUADs and 147 matched NCs (**Figure 5**), with AUCs ranging from 0.579 to 0.633. These results indicated the consistency of the results from the protein array and from ELISA. Moreover, the specificities of the five IgM indicators in LUADs were investigated in 72 LUSC and 44 SCLC patients. Only the titer of IgM autoantibodies against JAK2 in LUSC patients was higher than in NCs (*p* = 0.046) (**Figure 5A**). Moreover, the diagnostic performance of each IgM autoantibody in LUADs of the validation cohort was assessed using a series of evaluation indices in diagnostic experiments (**Table 2**). Regrettably, the individual IgM autoantibodies showed poor diagnostic value, with sensitivities ranging from 9.52% to 17.01% when the specificities were set higher than 90%. The diagnostic accuracies only ranged from 51.70% to 54.76%. Besides, the levels of the five IgM autoantibodies in LUADs with different clinical characteristics (clinical stage, gender, age, smoking history, and condition of lymph node metastasis, and distant metastasis) were stratified and analyzed in **Supplementary Figure S1**.

**Table 2:** Diagnostic value of five IgM autoantibodies in the validation cohort for the detection of lung adenocarcinoma (LUAD).

IgM Autoantibodies	Cutoff	Sen (%)	Spe (%)	AUC (95%CI)	PPV (%)	NPV (%)	+LR	−LR	Accuracy (%)
TSHR	0.432	9.52	93.88	0.599 (0.534–0.664)	60.87	50.92	1.556	0.964	51.70
ERBB2	0.381	17.01	92.52	0.613 (0.548–0.677)	69.44	52.71	2.274	0.897	54.76
Survivin	0.324	14.97	90.48	0.579 (0.514–0.644)	61.11	52.11	1.572	0.940	52.72
PIK3CA	0.363	14.29	93.20	0.601 (0.537–0.666)	67.74	52.09	2.101	0.920	53.74
JAK2	0.341	14.29	93.20	0.633 (0.570–0.697)	67.74	52.09	2.101	0.920	53.74

The optical density (OD) value at the maximum Youden’s index while specificity is more than 90% was defined as the cutoff value.

Sen, sensitivity; Spe, specificity; AUC, area under the receiver operating characteristic curve; CI, confidence interval; LUAD, lung adenocarcinoma; OD, optical density; PPV, positive predictive value; NPV, negative predictive value; +LR, positive likelihood ration; −LR, negative likelihood ration.

Since IgG autoantibodies to tumor-associated antigens have been frequently investigated, we also conducted an analysis on the five IgG autoantibodies. As shown in **Supplementary Figure S2**, three IgG autoantibodies (ERBB2, JAK2, and PIK3CA) were found to be significantly higher in LUADs than in NCs (AUC > 0.05, *p* < 0.05).

### Construction of a Diagnostic Model Combining IgM Autoantibodies and CEA

**Figure 6:**
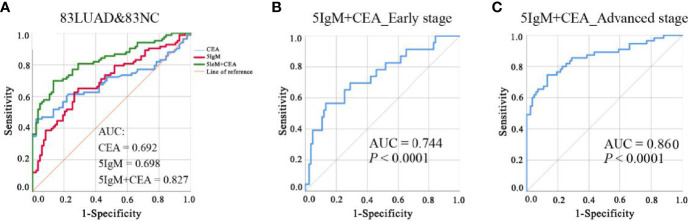
**(A)** ROC analysis of CEA alone, five IgM autoantibodies alone, and the combination of five IgM and CEA for the detection of LUAD from NC. **(B, C)**. ROC analysis of the diagnostic model for LUAD patients at different stages of the disease. AUC, area under the receiver operating characteristic curve; CEA, carcinoembryonic antigen; LUAD, lung adenocarcinoma; NC, normal control; ROC, receiver operating characteristic.

Given the insufficiency of a single IgM autoantibody for LUAD detection, we selected and matched the populations (83 LUADs and 83 NCs) with CEA results from the validation cohort to construct a diagnostic model that combines candidate IgM autoantibodies and CEA. Binary logistic regression was adopted to combine the IgM autoantibodies with CEA. It was found that the AUC of CEA alone was only 0.692 and that of the five IgM autoantibodies (combined) was 0.698. However, the AUC of the model that included both five IgM autoantibodies and CEA reached 0.827 (**Figure 6A**). This model effectively improved the diagnostic performance in LUADs. Moreover, the AUCs of this model in early- and advanced-stage LUADs reached 0.774 and 0.860, respectively (**Figures 6B, C**). The formula for the diagnostic model was as follows: *P* = 1/[1 + Exp(−(1.655*ERBB2 + 7.862*JAK2 − 10.285*TSHR + 17.135*PIK3CA − 11.294*Survivin + 0.299*CEA − 1.899)].

### Application of the Diagnostic Model Combining IgM Autoantibodies and CEA in Stratified Diagnosis of LUAD

LUADs were stratified according to their clinical characteristics of tumor stage, lymph node metastasis, and distant metastasis. ROC analysis and evaluation of the diagnostic performance were performed for every subgroup and in NCs (**Table 3**). The results revealed that the LUADs in every subgroup could be significantly distinguished from NCs based on this diagnostic model. In addition, the AUCs in LUADs with early-stage disease, lymph node metastasis (−), and distant metastasis (−) were higher than 0.7 (0.744, 0.804, and 0.769, respectively). Moreover, the AUCs in LUADs with advanced-stage disease, lymph node metastasis (+), and distant metastasis (+) reached 0.85 (0.860, 0.843, and 0.892, respectively). Notably, the accuracy of the model in early-stage LUADs was up to 83.02%. Interestingly, the predictive probability for LUAD derived by the model increased with the higher aggressiveness of the tumor [median: early stage (0.557) *vs*. advanced stage (0.803) and without lymph node metastasis (0.565) *vs*. with lymph node metastasis (0.900) *vs*. without distant metastasis (0.557) *vs*. with distant metastasis (0.905), all *p* < 0.05] (**Table 3**).

**Table 3:** Diagnostic value of the model combining five IgM autoantibodies and CEA for LUAD patients with different disease stages.

Group	Median	*p*-value	Sen (%)	Spe (%)	AUC (95%CI)	PPV (%)	NPV (%)	+LR	−LR	Accuracy (%)
All	0.716		56.63	93.98	0.827 (0.765–0.890)	90.38	68.42	9.407	0.462	75.30
**Stage**										
Early	0.557	0.004	39.13	95.18	0.744 (0.625–0.864)	69.23	84.95	8.118	0.640	83.02
Advanced	0.803		65.45	92.77	0.860 (0.793–0.928)	85.71	80.21	9.053	0.372	81.88
**Lymph node metastasis**										
No	0.565	0.017	43.75	92.77	0.804 (0.712–0.896)	70.00	81.05	6.051	0.606	79.13
Yes	0.900		63.83	96.39	0.843 (0.764–0.921)	90.91	82.47	17.681	0.375	84.62
**Distant metastasis**										
No	0.557	0.002	44.19	93.98	0.769 (0.679–0.859)	79.17	76.47	7.341	0.594	76.98
Yes	0.905		67.65	96.39	0.892 (0.816–0.967)	88.46	87.91	18.740	0.334	88.03

Median, median of the predictive probability value; Sen, sensitivity; Spe, specificity; AUC, area under the receiver operating characteristic curve; CI, confidence interval; LUAD, lung adenocarcinoma; PPV, positive predictive value; NPV, negative predictive value; +LR, positive likelihood ration; −LR, negative likelihood ration.

The diagnostic value of the model that included three IgG autoantibodies (ERBB2, JAK2, and PIK3CA) and CEA for LUADs is exhibited in **Supplementary Table S2**. The diagnostic performance of the model combining three IgG autoantibodies and CEA (AUC = 0.781, sensitivity = 56.63%, specificity = 90.36%) (**Supplementary Table S2**) was a little lower than that of the model that included five IgM autoantibodies and CEA (AUC = 0.827, sensitivity = 56.63%, specificity = 93.98%) in 83 LUADs *vs*. 83 NCs (**Table 3**). Interestingly, the model that included five IgM autoantibodies and CEA possessed great diagnostic value in early-stage LUAD (AUC = 0.744, *p* < 0.05) (**Table 3**), but the model that included three IgG autoantibodies and CEA had no diagnostic value in early-stage LUAD (AUC = 0.549, *p* > 0.05) (**Supplementary Table S2**).

## Discussion

In recent studies, the EarlyCDT-Lung test has achieved remarkable results in distinguishing LC patients from high-risk subjects. The panel, which includes seven IgG autoantibodies, demonstrated sensitivity and specificity values for newly diagnosed LC of about 40% and 90%, respectively (22–24). Regrettably, this detection method has not been widely used clinically due to its limitation of low sensitivity. Theoretically, IgM autoantibodies, as the first antibodies produced by the immune response, may be more suitable for screening indicators of early cancer diagnosis. The study by Fitzgerald et al. found that the IgM autoantibodies against CADM1, ICLN, SEC16, and ZNF768 were increased in the serum of colorectal cancer patients and demonstrated that these IgM autoantibodies are potential biomarkers for colorectal cancer (25). However, research works on the screening of autoantibody biomarkers for LC diagnosis and treatment have mainly focused on IgG autoantibodies (26–28). Notably, a study has shown that IgM autoantibodies play a crucial part in the immunosurveillance mechanisms against malignant epithelial cells (29). There is an urgent need to explore the expressions of IgM autoantibodies in LC patients for the screening of indicators that have the potential to identify malignant lesions at an earlier stage.

A fundamental target in cancer research is to understand the mechanisms of cell transformation. Cancer driver genes are involved in tumorigenesis and development across cancer types (30). Hence, IgM autoantibodies against proteins encoded by cancer driver genes might be potential biomarkers for the detection of LUAD. To test this hypothesis, we customized the protein array based on the 138 cancer-driving genes revealed in the genomic landscapes of common forms of human cancer by Vogelstein et al. (31) and endeavored to explore the expressions of IgM autoantibodies in the sera of LUADs and NCs in order to screen for indicators with potential diagnostic value. In phase I of this study, there was a significantly higher IgM autoantibody response to 31 antigens in LUADs than in NCs. In addition, the results indicated that the top 5 IgM autoantibodies (TSHR, ERBB2, survivin, PIK3CA, and JAK2) were capable of identifying patients with LUAD from NC individuals, whether it is the small sample screening data of the protein chip or the large sample verification data of ELISA. Importantly, these indicators showed excellent specificity in LUADs, but without increasing in other subtypes of LC.

ERBB2 is a member of the epidermal growth factor (EGF) receptor family of receptor tyrosine kinases, and its IgG autoantibody has been extensively assessed as a potential diagnostic biomarker for gastric cancer (32) and breast cancer (33). In a recent study, the determination of autoantibodies to TSHR presented helpful in the diagnosis and management of patients with autoimmune thyroid disease (34). In our previous study, potential IgG autoantibodies were screened in 100 serum samples from LC patients and 50 sera from NCs using focused protein array based on cancer driver genes. Interestingly, higher levels of IgG autoantibodies against survivin, PIK3CA, and JAK2 were observed in LC patients than in NCs (19). Therefore, IgM autoantibodies to TSHR, ERBB2, survivin, PIK3CA, and JAK2 may also be used as potential biomarkers for LUAD.

In phase II of the present study, we found that single-autoantibody indicators always showed an insufficient diagnostic ability, as in previous studies (35–37). Moreover, the AUCs of the five IgM and CEA were only 0.698 and 0.692, respectively. However, combining the five IgM autoantibodies with CEA by logistic regression resulted in a significant improvement in the AUC, sensitivity, and specificity, as well as the positive predictive value (PPV) and negative predictive value (NPV) of the assay. Besides, it is worth noting that the diagnostic accuracy in early-stage LUADs reached 83.02%. The results were better than that of the currently used EarlyCDT-Lung test with an accuracy of about 68% (22, 23, 38). Therefore, integrating two different types of biomarkers tends to have better diagnostic performance than that of a panel of multiple indicators of a single type.

Interestingly, the diagnostic performance of the model with five IgM autoantibodies and CEA (AUC = 0.827) was a little higher than that of the model with three IgG autoantibodies and CEA (AUC = 0.781). Additionally, for early-stage LUAD, the model with five IgM autoantibodies and CEA showed great diagnostic value, but the model with three IgG autoantibodies and CEA had no diagnostic value. This would justify IgM autoantibodies as having the advantage of potentially identifying malignant lesions at an earlier stage than IgG autoantibodies, as reported in the study of Pedchenko et al. (39), where high titers of IgM lung cancer-associated autoantibodies in the serum of patients 2 years before clinical presentation were detected. The diagnostic model that combined IgM and CEA may be a great auxiliary diagnostic tool for LUAD, especially for early-stage LUAD. Meanwhile, the predictive probability value of the diagnostic model increased with the aggressiveness of LUAD, which indicated that the model may have certain predictive power for the condition of patients.

Our study displayed several novel features. Firstly, we adopted a new modality of a customized protein array based on cancer driver genes to improve the possibility of screening potential IgM autoantibodies, which yielded satisfactory results. Secondly, ELISA was applied to validate the candidate IgM autoantibodies in an independent validation cohort with the aim of identifying robust LUAD biomarkers. Thirdly, the model constructed with the combination of IgM autoantibodies and CEA improved the diagnostic value of the existing clinical auxiliary diagnostic method, which implied that multi-analyte tests could ameliorate the problem of the insufficient diagnostic value of biomarkers.

There were certain limitations in this retrospective study. In future work, we will longitudinally explore the levels of these IgM indicators using pre-diagnostic longitudinal sera from LUADs. Moreover, we will verify the performance of the diagnostic model in a larger sample cohort to evaluate its capability in the early stage of LUAD.

In conclusion, we performed a meaningful approach of a protein array assay to identify novel IgM autoantibodies for the detection of LUAD. The diagnostic model that combined five IgM autoantibodies and CEA may be a more accurate method for the early detection of LUAD and possess certain predictive power for the severity of tumor.

## Data Availability Statement

The original contributions presented in the study are included in the article/**Supplementary Material**. Further inquiries can be directed to the corresponding author.

## Ethics Statement

The studies involving human participants were reviewed and approved by the Medical Ethics Committee of Zhengzhou University. The patients/participants provided written informed consent to participate in this study. Written informed consent was obtained from individual(s) for the publication of any potentially identifiable images or data included in this article.

## Author Contributions

LD was responsible for study conception and design. LD and JZ provided administrative support. TW, LP, FL, XiZ, and XW contributed to the provision of study materials or patients. XuZ, JL, YW, ML, and DJ collected and organized data. XuZ analyzed and interpreted the data and wrote the manuscript. All authors contributed to the article and approved the final manuscript.

## Funding

This work was supported by the Leading Talents of Science and Technology Innovation in Henan Province (grant no. 20420051008), the National Natural Science Foundation of China (grant no. 8167291), the Major Project of Science and Technology in Henan Province (grant no. 16110311400), the Key Project of Discipline Construction of Zhengzhou University (grant no. XKZDQY202009), and the Project of Basic Research Fund of Henan Institute of Medical and Pharmacological Sciences (grant no. 2020BP0202).

## Conflict of Interest

The authors declare that the research was conducted in the absence of any commercial or financial relationships that could be construed as a potential conflict of interest.

## Publisher’s Note

All claims expressed in this article are solely those of the authors and do not necessarily represent those of their affiliated organizations, or those of the publisher, the editors and the reviewers. Any product that may be evaluated in this article, or claim that may be made by its manufacturer, is not guaranteed or endorsed by the publisher.

## Abbreviations

AIS, adenocarcinoma *in situ*; AUC, area under the receiver operating characteristic curve; BSA, bovine serum albumin; CA-125, cancer antigen 125; CEA, carcinoembryonic antigen; CI, confidence internal; CYFRA 21-1, cytokeratin-19 fragment; EGF, epidermal growth factor; ELISA, enzyme-linked immunosorbent assay; GO, Gene Ontology; HRP, horseradish peroxidase; KEGG, Kyoto Encyclopedia of Genes and Genomes; LC, lung cancer; LDCT, low-dose computed tomography; LUAD, lung adenocarcinoma; LUSC, lung squamous cell carcinoma; MIA, minimally invasive adenocarcinoma; NCs, normal controls; NSCLC, non-small cell lung carcinoma; OD, optical density; PBS, phosphate-buffered saline; PBST, phosphate-buffered saline containing 0.1% Tween 20; ROC, receiver operating characteristic; SCLC, small cell lung carcinoma; SNR, signal-to-noise ratio.
