# Supplementary material for: A Diagnostic Model With IgM Autoantibodies and Carcinoembryonic Antigen for Early Detection of Lung Adenocarcinoma
Source: Front Immunol. 2022 Jan 24;12:728853. doi: 10.3389/fimmu.2021.728853 (PMC8818794; doi:10.3389/fimmu.2021.728853)
Supplement: Supplementary file 1 [file DataSheet_1.docx]

**Supplementary Figures and Tables**

**Table S1.** The descriptions/functions of 31 overexpressed IgM autoantibodies by protein array in discovery cohort for LUAD detection.

| IgM autoantibodies | Gene name | Classification | Process | Core pathway | AUC(95%*CI*) | *P*^a^ | FC | *P*^b^ |
| --- | --- | --- | --- | --- | --- | --- | --- | --- |
| TSHR | Thyroid stimulating hormone receptor | Oncogene | Cell Survival | PI3K; MAPK | 0.758(0.677-0.839) | 0.000* | 1.24 | 0.000* |
| ERBB2 | Erb-b2 receptor tyrosine kinase 2 | Oncogene | Cell Survival | PI3K; RAS | 0.691(0.602-0.780) | 0.000* | 1.16 | 0.000* |
| Survivin | Survivin (Surv) | TSG/Oncogene | Cell Survival | Cell Death; Kinases; Nuclear Proteins; Signal Transduction | 0.682(0.593-0.772) | 0.000* | 1.16 | 0.000* |
| PIK3CA | Phosphatidylinositol 4,5-bisphosphate 3-kinase catalytic subunit alpha isoform | Oncogene | Cell Survival | PI3K | 0.673(0.583-0.763) | 0.000* | 1.15 | 0.001* |
| JAK2 | Janus kinase 2 | Oncogene | Cell Survival | STAT | 0.665(0.573-0.756) | 0.001* | 1.13 | 0.002* |
| TSC1 | Tuberous sclerosis 1 | TSG | Cell Survival | PI3K | 0.659(0.567-0.751) | 0.001* | 1.11 | 0.002* |
| MED12 | Mediator complex subunit 12 | Oncogene | Cell Survival | Cell Cycle/Apoptosis; TGF-b | 0.657(0.565-0.749) | 0.002* | 1.14 | 0.002* |
| CTNNB1 | Catenin beta 1 | Oncogene | Cell Fate | APC | 0.650(0.557-0.743) | 0.003* | 1.12 | 0.001* |
| FGFR2_1 | Fibroblast growth factor receptor 2 | Oncogene | Cell Survival | PI3K; RAS ; STAT | 0.645(0.553-0.737) | 0.004* | 1.13 | 0.010* |
| RNF43 | Ring finger protein 43 | TSG | Cell Fate | APC | 0.640(0.547-0.733) | 0.005* | 1.12 | 0.008* |
| EGFR | Epidermal growth factor receptor | Oncogene | Cell Survival | PI3K; RAS | 0.639(0.546-0.733) | 0.005* | 1.13 | 0.005* |
| FOXL2 | Forkhead box L2 | Oncogene | Cell Fate | TGF-β | 0.633(0.540-0.727) | 0.007* | 1.09 | 0.013* |
| SRSF2 | Serine and arginine rich splicing factor 2 | Oncogene | Cell Fate | Transcriptional Regulation | 0.632(0.538-0.726) | 0.008* | 1.08 | 0.012* |
| IMP1 | Imprintor 1 | RNA-binding | RNA-binding | Nuclear Proteins | 0.628(0.534-0.722) | 0.010* | 1.12 | 0.020* |
| APC | Adenomatous polyposis coli | TSG | Cell Fate | APC | 0.624(0.530-0.718) | 0.012* | 1.12 | 0.007* |
| ALK | Anaplastic lymphoma receptor tyrosine kinase | Oncogene | Cell Survival | PI3K; RAS | 0.623(0.529-0.717) | 0.013* | 1.10 | 0.043* |
| SMO | Smoothened, frizzled class receptor | Oncogene | Cell Fate | HH | 0.623(0.528-0.718) | 0.013* | 1.08 | 0.039* |
| KLF4 | Kruppel-like factor 4 | Oncogene | Cell Fate | Transcriptional Regulation; WNT | 0.622(0.527-0.717) | 0.014* | 1.09 | 0.004* |
| PTPN11 | Protein tyrosine phosphatase, non-receptor type 11 | Oncogene | Cell Survival | RAS | 0.619(0.525-0.714) | 0.016* | 1.10 | 0.144 |
| FGFR2_2 | Fibroblast growth factor receptor 2 | Oncogene | Cell Survival | PI3K; RAS ; STAT | 0.619(0.524-0.713) | 0.017* | 1.11 | 0.023* |
| PDGFRA | Platelet-derived growth factor receptor, alpha polypeptide | Oncogene | Cell Fate | PI3K; RAS | 0.618(0.523-0.712) | 0.018* | 1.11 | 0.018* |
| SPOP | Speckle-type POZ protein | Oncogene | Cell Fate | Chromatin Modification; HH | 0.613(0.518-0.708) | 0.023* | 1.14 | 0.126 |
| HIST1H3B | Histone cluster 1, H3b | Oncogene | Cell Fate | Chromatin Modification | 0.611(0.516-0.706) | 0.026* | 1.10 | 0.028* |
| cMyc | Myelocytomatosis | Oncogene | Cell Survival | Cell Cycle/Apoptosis | 0.610(0.516-0.704) | 0.027* | 1.11 | 0.026* |
| FGFR3 | Fibroblast growth factor receptor 3 | Oncogene | Cell Survival | PI3K; RAS ; STAT | 0.605(0.509-0.700) | 0.035* | 1.11 | 0.061 |
| IKZF1 | IKAROS family zinc finger 1 | TSG | Cell Fate | Transcriptional Regulation | 0.602(0.506-0.697) | 0.041* | 1.11 | 0.111 |
| NFE2L2 | Nuclear factor (erythroid-derived 2)-like 2 | Oncogene | Cell Survival | Cell Cycle/Apoptosis | 0.601(0.507-0.696) | 0.041* | 1.10 | 0.016* |
| CDC73 | Cell division cycle 73 | TSG | Cell Survival | Cell Cycle/Apoptosis | 0.600(0.504-0.696) | 0.044* | 1.05 | 0.099 |
| JAK3 | Janus kinase 3 | Oncogene | Cell Survival | STAT | 0.599(0.504-0.695) | 0.046* | 1.10 | 0.050* |
| KIT | KIT proto-oncogene, receptor tyrosine kinase | Oncogene | Cell Survival | PI3K; RAS; STAT | 0.598(0.503-0.694) | 0.048* | 1.08 | 0.042* |
| EZH2 | Enhancer of zeste homolog 2 | Oncogene | Cell Fate | Chromatin Modification | 0.598(0.502-0.694) | 0.050* | 1.05 | 0.040* |

AUC: area under the receiver operating characteristic curve; CI: confidence interval; FC: fold change; LUAD: lung adenocarcinoma; TSG: Tumor Suppressor Gene

**Table S2** Diagnostic values of the model with three IgG autoantibodies (ERBB2/JAK2/PIK3CA) and CEA for LUAD patients with different stages

| Group | Median | *P* | Sen (%) | Spe (%) | AUC (95%*CI*) | PPV (%) | NPV (%) | +LR | -LR | Accuracy (%) |
| --- | --- | --- | --- | --- | --- | --- | --- | --- | --- | --- |
| All | 0.607 |  | 56.63 | 90.36 | 0.781 (0.708-0.854) | 85.45 | 67.57 | 5.874 | 0.480 | 73.50 |
| **Stage** | | | | | | | | | | |
| Early | 0.380 | <0.0001 | 17.39 | 96.39 | 0.549 (0.390-0.708) | 57.17 | 80.81 | 4.187 | 0.857 | 79.25 |
| Advanced | 0.672 |  | 70.91 | 90.36 | 0.880 (0.822-0.938) | 82.98 | 82.42 | 7.356 | 0.322 | 82.61 |
| **Lymph node metastasis** | | | | | | | | | | |
| No | 0.423 | <0.001 | 40.63 | 91.57 | 0.628 (0.495-0.762) | 65.01 | 80.00 | 4.820 | 0.648 | 77.40 |
| Yes | 0.758 |  | 68.09 | 90.36 | 0.875 (0.809-0.941) | 80.00 | 83.34 | 7.063 | 0.353 | 82.31 |
| **Distant metastasis** | | | | | | | | | | |
| No | 0.466 | <0.001 | 41.86 | 90.36 | 0.674 (0.562-0.787) | 69.23 | 75.00 | 4.342 | 0.643 | 73.81 |
| Yes | 0.726 |  | 73.53 | 90.36 | 0.891 (0.824-0.957) | 75.75 | 89.29 | 7.628 | 0.293 | 85.47 |

Median: median of predictive probability value; Sen: sensitivity, Spe: specificity, AUC: area under the receiver operating characteristic curve; *CI*: confidence interval; LUAD: lung adenocarcinoma; PPV: positive predictive value; NPV: negative predictive value; +LR: positive likelihood ration; -LR: negative likelihood ration


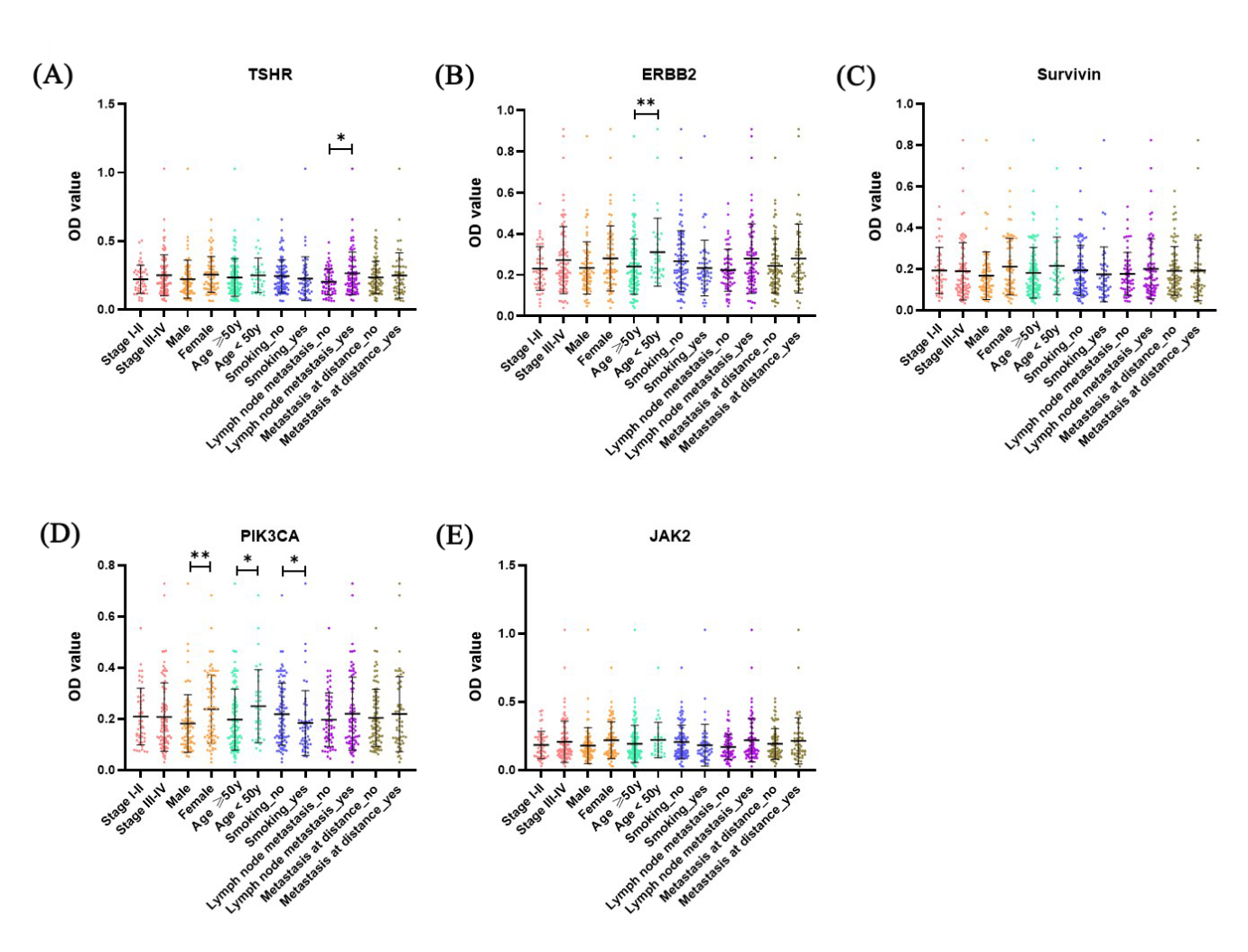


**Figure S1.** The expression of the top five IgM autoantibodies in LUADs with different clinical characteristics in validation cohort by ELISA. LUAD: lung adenocarcinoma; OD: optical density; ***P* < 0.01, **P* < 0.05


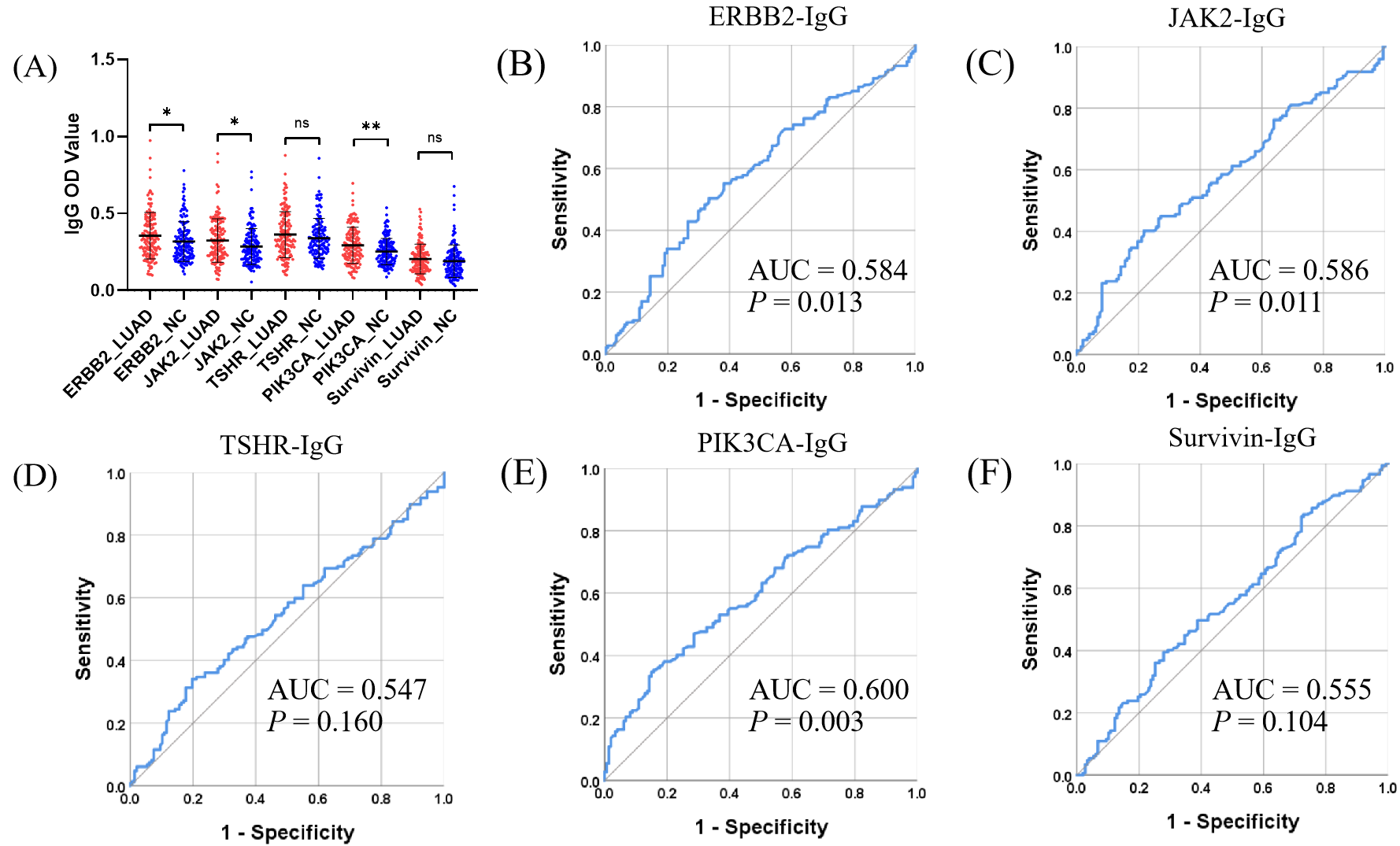


**Figure S2.** The differential expression and ROC analysis (LUAD and NC) of the five IgG autoantibodies in validation cohort by ELISA. ***P* < 0.01, **P* < 0.05, ^ns^*P* > 0.05; AUC: area under the receiver operating characteristic curve; LUAD: lung adenocarcinoma; LUSC: lung squamous cell carcinoma; NC: normal control; OD: optical density; ROC: receiver operating characteristic; SCLC: small cell lung carcinoma
